# Breastfeeding Duration and Development of Dysglycemia in Women Who Had Gestational Diabetes Mellitus: Evidence from the GUSTO Cohort Study

**DOI:** 10.3390/nu13020408

**Published:** 2021-01-28

**Authors:** Sumali S. Hewage, Xin Yu Hazel Koh, Shu E. Soh, Wei Wei Pang, Doris Fok, Shirong Cai, Falk Müller-Riemenschneider, Fabian Yap, Kok Hian Tan, Mei Chien Chua, Sok Bee Lim, Keith M. Godfrey, Marjorelee T. Colega, Yap-Seng Chong, Shiao-Yng Chan, Joanne Yoong, Mary F. F. Chong

**Affiliations:** 1 Saw Swee Hock School of Public Health, National University of Singapore, Singapore 117549, Singapore; sumali_hewage@u.nus.edu (S.S.H.); hazelkohxinyu@gmail.com (X.Y.H.K.); ephmf@nus.edu.sg (F.M.-R.); 2 Singapore Institute for Clinical Sciences, Agency for Science, Technology and Research (ASTAR), 30 Medical Drive, Singapore 117609, Singapore; SOH_Shu_E@hpb.gov.sg (S.E.S.); cai_shirong@sics.a-star.edu.sg (S.C.); marjorelee_colega@sics.a-star.edu.sg (M.T.C.); obgcys@nus.edu.sg (Y.-S.C.); obgchan@nus.edu.sg (S.-Y.C.); 3 Department of Obstetrics and Gynaecology, Yong Loo Lin School of Medicine, National University of Singapore, National University Health System, Singapore 119228, Singapore; obgpww@nus.edu.sg (W.W.P.); obglnld@nus.edu.sg (D.F.); 4 Berlin Institute of Health (BIH), Charité-Universitätsmedizin Berlin, 10178 Berlin, Germany; 5 Duke-NUS Medical School, 8 College Road, Singapore 169857, Singapore; fabian.yap.k.p@singhealth.com.sg (F.Y.); tan.kok.hian@singhealth.com.sg (K.H.T.); or Chua.Mei.Chien@kkh.com.sg (M.C.C.); 6 Department of Paediatrics, KK Women’s and Children’s Hospital, 100 Bukit Timah Road, Singapore 229899, Singapore; 7 Lee Kong Chian School of Medicine, Nanyang Technological University, 11 Mandalay Road, Singapore 308232, Singapore; 8 Department of Maternal Fetal Medicine, KK Women’s and Children’s Hospital, 100 Bukit Timah Road, Singapore 229899, Singapore; 9 Department of Neonatology, KK Women’s and Children’s Hospital, Singapore 229899, Singapore; 10 Department of Child Development, KK Women’s & Children’s Hospital, Singapore 229899, Singapore; Lim.Sok.Bee@kkh.com.sg; 11 Medical Research Council Lifecourse Epidemiology Unit, Southampton SO16 6YD, UK; kmg@mrc.soton.ac.uk; 12 National Institute for Health Research Southampton Biomedical Research Centre, University of Southampton and University Hospital Southampton National Health Service Foundation Trust, Southampton SO16 6YD, UK; 13 Center for Economic and Social Research, University of Southern California, Los Angeles, CA 90089, USA; joanne.yoong@gmail.com

**Keywords:** breastfeeding, gestational diabetes, type 2 diabetes risk, prediabetes risk

## Abstract

(1) Background: Breastfeeding has been shown to support glucose homeostasis in women after a pregnancy complicated by gestational diabetes mellitus (GDM) and is potentially effective at reducing long-term diabetes risk. (2) Methods: Data from the Growing Up in Singapore Towards healthy Outcomes (GUSTO) study were analyzed to understand the influence of breastfeeding duration on long-term dysglycemia (prediabetes and diabetes) risk in women who had GDM in the index pregnancy. GDM and dysglycemia four to seven years postpartum were determined by the oral glucose tolerance test (OGTT). A Poisson regression model with a robust error variance was used to estimate incidence rate ratios (IRRs) for dysglycemia four to seven years post-delivery according to groupings of the duration of any breastfeeding (<1, ≥1 to <6, and ≥6 months). (3) Results: Women who had GDM during the index pregnancy and complete breastfeeding information and OGTT four to seven years postpartum were included in this study (*n* = 116). Fifty-one women (44%) had postpartum dysglycemia. Unadjusted IRRs showed an inverse association between dysglycemia risk and ≥1 month to <6 months (IRR 0.91; 95% confidence interval [CI] 0.57, 1.43; *p* = 0.68) and ≥6 months (IRR 0.50; 95% CI 0.27, 0.91; *p* = 0.02) breastfeeding compared to <1 month of any breastfeeding. After adjusting for key confounders, the IRR for the ≥6 months group remained significant (IRR 0.42; 95% CI 0.22, 0.80; *p* = 0.008). (4) Conclusions: Our results suggest that any breastfeeding of six months or longer may reduce long-term dysglycemia risk in women with a history of GDM in an Asian setting. Breastfeeding has benefits for mothers beyond weight loss, particularly for those with GDM.

## 1. Introduction

Gestational diabetes mellitus (GDM) is associated with increased risk of type 2 diabetes mellitus (T2DM) development [1,2]. It has been suggested that hyperglycemia, which arises primarily due to the effects of placental hormones during pregnancy, results in a lasting deterioration of insulin sensitivity, leading to the onset of T2DM, typically years after delivery [1,3]. Another explanation is that both diseases have common risk factors such as increased body mass index (BMI), advanced age, and a family history of T2DM [2]. Relative to women of Western ethnicity, Asian women are at higher risk for GDM [4] and T2DM [5]. In Singapore, the Growing Up in Singapore Towards healthy Outcomes (GUSTO) study found one in five pregnant participants had GDM [6]. However, compared to a similarly affluent country such as the United States, this is a higher prevalence [7] and it is also above the estimated global prevalence of 13.4% in 2019 [8]. Understanding the modifiable risk factors or behaviors that can influence the risk of developing T2DM in women with a history of GDM in an Asian setting is therefore of pressing interest.

Breastfeeding is widely recommended by global health organizations [9,10,11] and public health initiatives worldwide [12]. In addition to benefits for children, breastfeeding has been generally associated with reducing the risk of diseases, including breast cancer and T2DM [13,14,15,16,17]. The underlying mechanism of how breastfeeding may reduce long-term metabolic risk is not well understood [18,19,20,21,22]. However, the benefits of breastfeeding have been related to two key physiological changes. First, obesity is an independent modifiable risk factor for T2DM risk [23]. Breastfeeding is a high-energy demand task, primarily met by increased glycogenolysis and improved insulin sensitivity, which is an important metabolic adaptation [20,21,24,25,26,27]. Therefore, breastfeeding may act as an intervention that supports the return to an optimal healthy weight in the first postpartum year [28,29]. Second, the effect of prolactin on the initiation and maintenance of breast milk production and on glucose homeostasis by stimulating insulin release is well-recognized [30,31,32]. This proposed mechanism suggests that prolactin promotes more optimal blood glucose regulation and lipid metabolism and improves insulin sensitivity in the postpartum period, as the use of glucose for lactogenesis results in lower glucose and insulin concentrations [33,34]. It is possible that a combination of the effects described above results in breastfeeding aiding the course of healthier glycemic homeostasis. 

Recent meta-analyses of observational studies suggested that in women with prior GDM, longer durations of breastfeeding significantly reduce the risk of T2DM. Tanase-Nakao et al. (2017) reported that longer lactation (more than 4 and up to 12 weeks) of any intensity reduced diabetes risk compared to the shorter-lactation group [35]. Moreover, Ma et al. reported positive effects from long-term breastfeeding of any intensity to reduce the risk of dysglycemia in women after GDM pregnancy, particularly in long-term follow-up studies [36]. However, the studies included in the analyses were primarily conducted in Western populations [35,36].

To the best of our knowledge, few studies overall have examined the relationship between breastfeeding and the risk of dysglycemia in women with a history of GDM in an Asian setting. Moreover, no long-term follow-up study has yet ascertained whether breastfeeding reduces diabetes and prediabetes (here collectively termed dysglycemia) risk. To address this gap, we aimed to evaluate the association between breastfeeding and the development of dysglycemia after GDM using uniquely detailed longitudinal data from participants in the Growing Up in Singapore Towards healthy Outcomes (GUSTO) study. In this study, we hypothesized that a longer duration of any breastfeeding would reduce the risk of dysglycemia four to seven years post-delivery in women with a history of GDM.

## 2. Materials and Methods

### 2.1. Study Population

This study was conducted using data from women diagnosed with GDM during the index pregnancy who were participating in the GUSTO mother–offspring cohort study. The primary objective of the birth cohort was to evaluate the pathways by which exposures in the early developmental stages lead to later metabolic adversity. Briefly, pregnant women in the first trimester of pregnancy who were seeking antenatal care at KK Women’s and Children’s Hospital (KKH) or the National University Hospital (NUH), the two largest public maternity units in Singapore, were recruited between June 2009 and September 2010. Key eligibility criteria included being 18 to 46 years of age, belonging to one of the three main ethnic groups of Singapore (Chinese, Malay, or Indian), and being either a citizen or a permanent resident of Singapore. Women diagnosed with type 1 diabetes mellitus or treated with chemotherapy or psychotropic drugs were excluded. Ethical approval was obtained from the National Healthcare Group Domain Specific Review Board and the SingHealth Centralised Institutional Review Board. All women provided written informed consent. More detailed information about the GUSTO project is extensively reported elsewhere [37].

**Figure 1:**
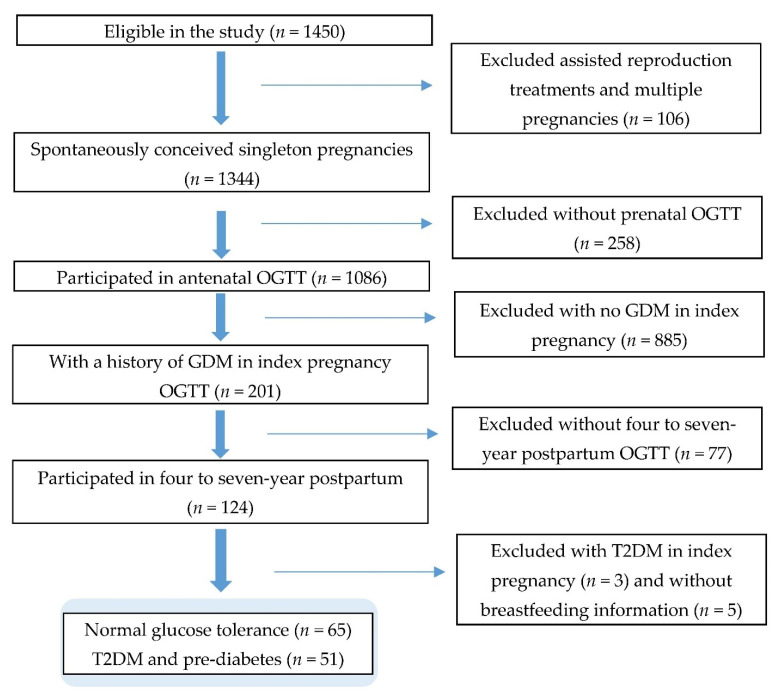
Study flow diagram.

For this study, we included women who completed the oral glucose tolerance test (OGTT) on two occasions—around 26 to 28 weeks’ gestation and four to seven years after the index pregnancy—and provided breastfeeding information. Women were diagnosed with GDM by the WHO 1999 criteria (fasting glucose ≥ 7.0 mmol/L and/or 2 h glucose ≥ 7.8 mmol/L after a 75 g glucose load) [38] in the antenatal OGTT. Women with likely pre-existing T2DM (self-reported or medically diagnosed in the prenatal OGTT with fasting glucose ≥ 7.0 mmol/L or 2 h glucose ≥ 11.1 mmol/L), those who underwent assisted reproduction treatments, and those with multiple pregnancies were excluded (Figure 1).

### 2.2. Data Collection

Quantitative data were collected between study enrolment and seven-year postpartum follow-up [37].

#### 2.2.1. Ascertainment of T2DM and Prediabetes

The main outcome variables were the incidence of T2DM and prediabetes four to seven years postpartum, as evaluated by a 75 g OGTT. T2DM was diagnosed by a fasting blood glucose of ≥7.0 mmol/L (126 mg/dL) or 2 h plasma glucose of ≥11.1 mmol/L (200 mg/dL), while impaired glucose tolerance (fasting blood glucose < 7.0 mmol/L (126 mg/dL) with 2 h plasma glucose ≥ 7.8 and <11.1 mmol/L (140 and 200 mg/dL)) and impaired fasting glucose (6.1–6.9 mmol/L (110–125 mg/dL) and 2 h plasma glucose < 7.8 mmol/L (<140 mg/dL)) were diagnosed by the 2006 WHO [39] guidelines.

Even without established T2DM, women with prediabetes (either impaired glucose tolerance or impaired fasting glucose) were considered to have above normal blood glucose concentrations and a prediagnosis of future diabetes. T2DM and prediabetes were grouped as the dysglycemia category, while those with normal blood glucose levels were placed under the normal glucose tolerance (NGT) category.

#### 2.2.2. Assessment of Breastfeeding Duration

Breastfeeding information about infant feeding and the age at which breastfeeding ceased were collected from GUSTO participants using interviewer-administered questionnaires starting from 3 weeks postpartum to every 3 months until 12 months postpartum [40]. Based on published data and current WHO recommendations, breastfeeding was grouped into durations of <1 month, ≥1 month to <6 months, and ≥6 months [41,42]. The women who did not breastfeed were included in the <1 month group.

Measurement of covariates: Based on published studies [18,43,44], the following variables were included in the statistical analysis. At recruitment (<14 weeks’ gestation), interview-administered questionnaires were conducted to collect demographic details, including ethnicity (Chinese, Malay, and Indian), highest education (secondary or below, diploma/technical education, and university or higher), monthly household income (≤S$6000 and >S$6000) [45], and parity (0, 1, and ≥2). In addition, a self-reported family history of T2DM (yes and no) and GDM history prior to the index pregnancy (yes and no) were collected. At 26 to 28 weeks’ gestation, the women were asked to record their dietary intake in a three-day food diary. Moreover, a 24 h dietary recall was collected by trained clinical staff. Diet quality was assessed using the healthy eating index for Singapore pregnant women (HEI-SGP) [46]. Data on the frequency and duration of three different intensities of physical activity during the year before pregnancy and the first six months of pregnancy were collected. Based on the International Physical Activity Questionnaire (IPAQ) short form [47], the energy expended on physical activity was calculated and expressed as metabolic equivalent (MET)/week (<600, 600–3000, and >3000 MET/week) [48]. Information about tobacco smoking exposure pre-pregnancy and during pregnancy was collected (exposed and not exposed). The mother’s age was collected at child delivery (≤30, 31–35, and ≥36 years). The pre-pregnancy body mass index (BMI) was calculated using the self-reported pre-pregnancy weight at recruitment and the height measured using research-quality calibrated instruments at the 26-week antenatal visit. The type of GDM treatment was retrieved from the participant’s medical records (diet only, insulin, and unknown, or none). The mother’s height and weight information was collected 18 months postpartum and yjr waist circumference at the postpartum 48-month visit.

### 2.3. Statistical Analysis

Univariate analyses were conducted to describe and compare the demographic factors, health, anthropometric measurements, lifestyle factors, and breastfeeding behaviors between dysglycemia and Normal glucose tolerance (NGT) groups. Group differences were evaluated using Student’s *t*-test or the Wilcoxon rank-sum test and ANOVA for continuous variables and chi-square and Fisher’s exact tests for categorical variables. Variables were also assessed for collinearity.

Adjusted and unadjusted multivariate analyses were undertaken using a Poisson regression model with a robust error variance [49] to estimate an incidence rate ratio (IRR) between breastfeeding and progression to dysglycemia four to seven years postpartum. Covariates were identified and considered for model building based on the literature and statistical significance for possible clinically important variables. Potential confounders were determined and included in the final regression model if there was a 10% change in the regression coefficient in one or more lactation groups. The model was adjusted for the mother’s age at delivery, parity at recruitment, a family history of diabetes at recruitment, previous GDM, the pre-pregnancy BMI, and tobacco smoking exposure. Statistically significant results were determined at *p <* 0.05. All point estimates were presented with 95% confidence intervals (CIs). Statistical analyses were performed using STATA software version 13.0 (StataCorp LP, College Station, TX, USA).

## 3. Results

The final statistical analyses were based on data for 116 women who had been diagnosed with GDM in the index pregnancy, had breastfeeding data, and had completed an OGTT four to seven years (median 5.1; interquartile range (IQR) 4.1–5.2 years) postpartum.

The characteristics of women who developed and those who did not develop dysglycemia four to seven years postpartum are shown in Table 1. Of the women tested, 51 (44.0%) had abnormal postpartum glucose tolerance test results, with 13 (11.2%) diagnosed with T2DM and 38 (32.8%) with prediabetes.

Compared to NGT women, women in the dysglycemia group were older, of higher parity (marginally significant), and more likely to have a family history of T2DM. In addition, they had a significantly higher pre-pregnancy BMI, postpartum BMI at the 18-month study visit, and waist circumference at the 48-month study visit compared to NGT women (Table 1).

Breastfeeding for six months or more was associated with the highest education attainment at recruitment (*p* < 0.05) and lower household income at recruitment (*p* = 0.02). Furthermore, a higher HEI-SGP score during pregnancy but before GDM diagnosis (*p* = 0.007) and no pre-pregnancy and during pregnancy tobacco smoking exposure (*p* < 0.05) showed significant association with any breastfeeding of six months or more.

The unadjusted model (Table 2) suggested that women with a history of GDM who breastfed for ≥1 to <6 months had a dysglycemia IRR of 0.91 (95% CI 0.57, 1.43; *p* = 0.687) compared to those who did not breastfeed or did so for <1 month. This relationship was augmented in women who breastfed for ≥6 months with an IRR of 0.50 (95% CI 0.27, 0.91; *p* = 0.02) compared to <1 month of breastfeeding duration. Compared to women who did not breastfeed or did so for <1 month, the covariate-adjusted IRR of women who breastfed for ≥1 month to <6 months was 0.67 (95% CI 0.41, 1.10; *p* = 0.117), while those who breastfed for ≥6 months had the lowest IRR of 0.42 (95% CI 0.22, 0.79; *p* = 0.08) (Table 2).

## 4. Discussion

This is the first study conducted on women in Singapore to understand the role of breastfeeding in reducing long-term T2DM and prediabetes risk following a diagnosis of GDM. Three different Asian ethnicities were studied with a postpartum follow-up period of up to seven years. Our analysis includes prospectively gathered potential confounders, based on empirical evidence that gestational age at diagnosis of gestational diabetes [50], parity [51], a family history of diabetes [52], the pre-pregnancy BMI [53], and tobacco smoking exposure [54] are either risk factors, preventive factors, or surrogates for T2DM risk.

We find that the protective effect of breastfeeding to reduce diabetes risk is associated with a longer breastfeeding duration, even after controlling for the mother’s age at delivery, parity at recruitment, a family history of diabetes at recruitment, previous GDM, the pre-pregnancy BMI, and perinatal tobacco smoking exposure, as supported.

Recently published meta-analyses have reported positive effects of long-term breastfeeding of any intensity to reduce diabetes risk in women with a history of GDM [35,36]. In addition, Feng et al. (2018) evaluated 13 observational studies and compared the effect of lactation with that of no lactation on diabetes risk reduction; they found a significant diabetes risk reduction in the lactation group [55]. The authors reported that exclusive lactation for more than six weeks and up to nine weeks postpartum showed an association with a lower risk of T2DM compared to exclusive formula feeding [55]. Still, the meta-analyses reported by Tanase-Nakao et al. (2017) and Feng et al. (2018) lacked prospective data of cohorts with long-term follow-up. Moreover, all three meta-analyses included limited evidence on breastfeeding duration and T2DM risk among women with a history of GDM and Asian origin [35,36,55]. In light of these limitations, this paper adds to and extends the existing body of evidence on breastfeeding in Asian populations, which to date, has focused primarily on only glycemic control within a narrow follow-up period after delivery.

Outside Asia, the beneficial effects of long-term breastfeeding on reducing the risk of dysglycemia in women with previous GDM have been reported in some observational studies [13,18,19,22,56,57,58] but found to be absent in others [59,60,61]. Previous studies in Asian populations have had mixed findings and have been limited by their duration. Kim et al. (2011) found no association between lactation and glycemic outcomes in Korean mothers. Still, they performed only a cross-sectional analysis of the early postpartum period of 6 to 12 weeks [62], which was too limited for the typical manifestation of dysglycemia. On the other hand, Yasuhi et al. (2017) reported that compared to low-intensity breastfeeding, high-intensity breastfeeding led to lower odds of developing abnormal glucose tolerance after the first year postpartum [21]. Our results further corroborate findings from the Shanghai Women’s Health Study, conducted in a predominantly Chinese population. Villegas et al. (2008) showed that after controlling potential confounders at the baseline, the increased duration of lifetime breastfeeding is inversely associated with T2DM risk after more than four years of follow-up [44]. No existing studies in Asia extended the investigation to determine the association of breastfeeding and T2DM risk in women with previous gestational diabetes [21,44,62].

This study was not designed to investigate the mechanisms by which breastfeeding reduces T2DM risk. However, while controlling for contemporaneous risk factors, we observed that breastfeeding benefits appear to persist in the long term. This may suggest that the effects of breastfeeding manifest in a more complex way than a purely short-term physiological response to breastfeeding. It may also offer additional intergenerational benefits through impeding the risk of metabolic disease and disrupting the ongoing vicious cycle of offspring obesity and a future risk of diabetes [63]. Future studies may investigate this further and potentially explore differential responses to breastfeeding based on non-modifiable factors that affect metabolism. For instance, one German study revealed that women positive for islet antibodies had the highest risk of T2DM and did not appear to benefit from the protective effect of breastfeeding. In contrast, women without islet antibodies who breastfed for more than three months had a reduced risk of T2DM [22].

A few limitations were noted. The small sample size and sample characteristics may limit the generalizability of the study findings. Moreover, the dysglycemia group included women with prediabetes and T2DM. As prediabetes is a potentially reversible condition, the inferences drawn here may differ from those that evaluate the risk of only one of the two categories (i.e., T2DM or prediabetes). Moreover, the study analysis was restricted to glycemic outcome data collected after the fourth year post-delivery. Therefore, the transient dysglycemia that had developed prior to this time has not been ascertained.

Of the 201 women who had GDM in the index pregnancy, only 124 had completed the four- to seven-year postpartum OGTT offered by the GUSTO study team and provided breastfeeding data. These women may have been more health conscious than women who were excluded from this study, which may have impacted the results due to self-selection bias. Subsequently, 38.3% of women who had GDM were excluded from the study.

Even though there is a possibility for existing non-response bias, comparison of the demographic information did not reveal any statistically significant differences between women who completed and who did not complete the postpartum OGTT and between women with and without breastfeeding data (Table S1). Moreover, there is the possibility of misclassification and social desirability bias. However, recall bias has been minimized by comprehensive and frequent data collection conducted postpartum. Although considerable effort was made to address confounding, possible residual confounding, primarily from socio-economic factors, may have influenced the effect of breastfeeding. This study did not account for variations in breastfeeding intensity or the impact of lifetime breastfeeding on glycemic outcomes.

Our study results using the WHO 1999 criteria for GDM diagnosis may not be generalizable to the current GDM population diagnosed using the WHO 2013 criteria, which uses different glucose thresholds in a three-time-point antenatal OGTT for diagnosis. Finally, this observational study cannot establish a causal relationship between breastfeeding and reduced diabetes risk.

## 5. Conclusions

In conclusion, this study of Asian women who had GDM showed an increasing inverse association between breastfeeding and diabetes risk, which was strongest in women who breastfed for at least six months and less in those who breastfed for a shorter duration. With effective strategies of extended maternity leave and employer assistance aiming to increase breastfeeding intensity and duration, public health programs are worth considering, especially in a population like Singapore with a high diabetes prevalence of 8.6% [64].

Pregnancy holds a unique opportunity to identify women at risk of abnormal insulin sensitivity in the long term through the diagnosis of GDM. Moreover, breastfeeding after pregnancy is a low-cost intervention that may improve future maternal cardio-metabolic risk. However, the causality between breastfeeding duration and the development of dysglycemia in women who had GDM is still unclear. There is a need to gather further scientific evidence on a larger and nationally representative GDM population diagnosed using the most updated criteria.

## Figures and Tables

**Table 1:** Characteristics of women who developed and did not develop dysglycemia 4 to 7 years postpartum after gestational diabetes (*n* = 116).

Characteristics	All (*n* = 116)	NGT (*n* = 65)	Dysglycemia (*n* = 51)	*p*-Value *
Glycemic activity *				
Antenatal fasting glucose, mmol/L, (median, IQR)	4.5 (4.2, 4.9)	4.4 (4.1, 4.8)	4.5 (4.2, 4.7)	0.26
Antenatal 2-h glucose, mmol/L, (median, IQR)	8.4 (8.1, 9.1)	8.3 (8.1, 9.1)	8.4 (8.1, 9.1)	0.84
Postpartum fasting glucose, mmol/L, (median, IQR)	4.9 (4.7, 5.3)	4.8 (4.6, 5.0)	5.2 (4.9, 5.4)	<0.05
Postpartum 2-h glucose, mmol/L, (median, IQR)	7.4 (6.4, 9.1)	6.5 (5.9, 6.9)	9.2 (8.4, 10.7)	<0.05
Socio-demographics				
Maternal age at delivery, years, (mean, SD)	33.3 (±4.8)	32.8 (±4.5)	33.9 (±5.2)	0.25
Ethnicity, *n* (%)				
Chinese	73 (62.9)	44 (67.7)	29 (56.9)	0.25
Malay	16 (13.8)	6 (9.2)	10 (19.6)	
Indian	27 (23.3)	15 (23.1)	12 (23.5)	
Education level, *n* (%)				
Secondary or below	24 (20.7)	11 (16.9)	13 (25.5)	0.33
Diploma/Technical education	43 (37.1)	23 (35.4)	20 (39.2)	
University or higher	49 (42.2)	31 (47.7)	18 (35.3)	
Household monthly income at recruitment, *n* (%)				
≤S$6000	73 (67.0)	39 (62.9)	34 (72.3)	0.30
>S$6000	36 (33.0)	23 (37.1)	13 (27.7)	
Parity at recruitment, *n* (%)				
0	45 (38.8)	27 (41.5)	18 (35.3)	0.06
1	50 (43.1)	31 (47.7)	19 (37.2)	
≥2	21 (18.1)	7 (10.8)	14 (27.5)	
Health				
Family history of diabetes at recruitment, *n* (%)				
No	101 (87.1)	61 (93.8)	40 (78.4)	0.01
Yes	15 (12.9)	4 (6.2)	11 (21.6)	
Treatment of GDM, *n* (%)				0.14
Diet only	102 (88.0)	59 (90.8)	43 (84.3)	
Insulin	7 (6.0)	2 (3.1)	5 (9.8)	
Unknown or none	7 (6.0)	4 (6.1)	3 (5.9)	
GDM in a previous pregnancy, *n* (%)				0.37
Nulliparous	45 (38.8)	27 (41.5)	18 (35.3)	
No or missing	60 (51.7)	34 (52.3)	26 (51.0)	
Yes	11 (9.5)	4 (6.2)	8 (13.7)	
Anthropometric measurements				
Pre-pregnancy BMI, kg/m^2^ (mean, SD)	23.7 (±4.2)	22.7 (±3.8)	25.0 (±4.3)	<0.05
Postpartum BMI, kg/m^2^ at 18-month visit, *n* (%)				<0.01
<23	40 (42.6)	27 (51.9)	13 (31.0)	
23 to <27.5	34 (36.2)	20 (38.5)	14 (33.3)	
≥27.5	20 (21.3)	5 (9.6)	15 (35.7)	
Postpartum waist circumference at 48-month visit, *n* (%)				0.01
<80 cm	31 (32.0)	23 (42.6)	8 (18.6)	
≥80 cm	66 (68.0)	31 (57.4)	35 (81.4)	
Lifestyle				
Healthy eating index for Singapore pregnant women (HEI-SGP), (mean, SD)	57.3 (±13.7)	58.3 (±12.4)	56.0 (±15.3)	0.37
Physical activity before index pregnancy, *n* (%)				0.62
<600 MET/week	11 (12.1)	6 (11.5)	5 (12.8)	
600–3000 MET/week	61 (67.0)	37 (71.2)	24 (61.5)	
>3000 MET/week	19 (20.9)	9 (17.3)	10 (25.7)	
Perinatal tobacco smoking exposure, *n* (%)				0.49
Exposed	38 (32.8)	23 (35.4)	15 (29.4)	
Not exposed	78 (67.2)	42 (64.6)	36 (70.6)	
Breastfeeding behaviors				
Any breastfeeding, *n* (%)				0.03
No or <1 month	21 (18.1)	9 (13.9)	12 (23.5)	
≥1 to <6 months	50 (43.1)	24 (36.9)	26 (51.0)	
≥6 months	45 (38.8)	32 (49.2)	13 (25.5)	

* p-Value for univariate analysis. Categorical variables were expressed in frequencies and percentages, while continuous variables were presented as the mean (SD), unless otherwise specified. GUSTO, Growing Up in Singapore Towards healthy Outcomes; GDM, gestational diabetes mellitus; NGT, normal glucose tolerance; IRR incidence rate ratio; IQR interquartile range; OGTT, oral glucose tolerance test; BMI, body mass index; MET, Metabolic equivalent. * Based on a two time-point 75 g OGTT.

**Table 2:** Association between breastfeeding and development of dysglycemia postpartum.

Variables	Unadjusted IRR (95% CI)	*p*-Value	Age-Adjusted IRR (95% CI)	*p*-Value	Covariate-Adjusted IRR * (95% CI)	*p*-Value
Any breastfeeding						
No or <1 month	Reference		Reference		Reference	
≥1 to <6 months	0.91 (0.57, 1.43)	0.687	0.90 (0.57, 1.44)	0.682	0.67 (0.41, 1.10)	0.117
≥6 months	0.50 (0.27, 0.91)	0.024	0.51 (0.28, 0.93)	0.030	0.42 (0.22, 0.79)	0.008

* The model was adjusted for the mother’s age at delivery, parity at recruitment, a family history of diabetes at recruitment, previous GDM, the pre-pregnancy BMI, and perinatal tobacco smoking exposure. IRR, incident rate ratio.

## Data Availability

The datasets generated and/or analysed during the current study are not publicly available due to an ethical restriction (patient confidentiality) which was imposed by the Centralised Institutional Review Board of SingHealth. Interested researchers may request the data by contacting P Mukkesh Kumar at Mukkesh_Kumar@sics.a-star.edu.sg.

## Supplementary Materials

The following are available online at https://www.mdpi.com/2072-6643/13/2/408/s1: Table S1: socio-demographic comparison between GUSTO women with a history of GDM by completion of a postpartum OGTT.
