# Supplementary material for: Breastfeeding Duration and Development of Dysglycemia in Women Who Had Gestational Diabetes Mellitus: Evidence from the GUSTO Cohort Study
Source: Nutrients. 2021 Jan 28;13(2):408. doi: 10.3390/nu13020408 (PMC7912373; doi:10.3390/nu13020408)
Supplement: Supplementary file 1 [file nutrients-13-00408-s001.pdf]

Supplement Table 1: Socio-demographic comparison between GUSTO women with a history of GDM by completion of postpartum OGTT.

**Socio-demographic characteristics**	**Complete OGTT data**	**In-complete OGTT data**	***p-value***
	**n=124 (61.7%)**	**n=77 (38.3%)**	
Maternal age at delivery, years, n (%)			0.528
≤30	27 (21.8)	16 (25.0)	
31-39	83 (66.9)	44 (68.7)	
≥40	14 (11.3)	4 (6.3)	
Ethnicity, n (%)			0.955
Chinese	78 (63.4)	48 (62.3)	
Malay	17 (13.8)	12 (15.6)	
Indian	28 (22.8)	17 (22.1)	
Education level, n (%)			0.455
Secondary or below	25 (20.2)	20 (26.3)	
Diploma/Technical education	47 (37.9)	23 (30.3)	
University or higher	52 (41.9)	33 (43.4)	
Household monthly income, n (%)			0.875
≤ S$6,000	78 (66.7)	47 (65.3)	
> S$6,000	39 (33.3)	25 (34.7)	
Parity at recruitment, n (%)			0.939
0	47 (37.9)	26 (40.6)	
1	53 (42.7)	26 (40.6)	
≥ 2	24 (19.4)	12 (18.7)	

Abbreviations: GUSTO Growing Up in Singapore Towards healthy Outcomes; GDM gestational diabetes mellitus; OGTT oral glucose tolerance test; GCE General Certificate of Education. Categorical variables were expressed in frequencies and percentages. Women with a history of GDM in the main GUSTO cohort: 201
